# Serial Mediation Roles of Perceived Stress and Depressive Symptoms in the Association Between Sleep Quality and Life Satisfaction Among Middle-Aged American Adults

**DOI:** 10.3389/fpsyg.2022.822564

**Published:** 2022-02-21

**Authors:** Yanxu Yang, Yendelela L. Cuffee, Betsy B. Aumiller, Kathryn Schmitz, David M. Almeida, Vernon M. Chinchilli

**Affiliations:** ^1^Department of Public Health Sciences, Penn State College of Medicine and Milton S. Hershey Medical Center, Hershey, PA, United States; ^2^College of Health Sciences, University of Delaware, Newark, DE, United States; ^3^Department of Human Development and Family Studies, Pennsylvania State University, University Park, PA, United States

**Keywords:** sleep quality, perceived stress, depressive symptom, life satisfaction, serial mediation

## Abstract

In this study, we used data from the second wave of Midlife in the United States (MIDUS) Study, MIDUS Biomarkers and MIDUS 3. We applied the serial mediation model to explore the serial mediating effects of perceived stress and depressive symptoms on the relationship between sleep quality and life satisfaction. A total of 945 participants were included in our study. The total indirect effect of sleep quality on life satisfaction through perceived stress, depressive symptoms and the combination of perceived stress and depressive symptoms accounted for within the overall model was 45.5%. At the intervention level, programs designed to improve the level of life satisfaction among adults should focus on perceived stress and depressive symptoms. The prevention of perceived stress and depression contributes to improving life satisfaction and wellbeing. The serial mediation results should be confirmed by further longitudinal study.

## Introduction

Sleep is an important component of every individual's overall health and wellbeing. Sleep quality is a critical indicator that measures how well people sleep. Studies from different countries around the world showed that the prevalence of sleep problems, including insomnia, sleep apnea, restless legs syndrome, and narcolepsy, ranges from 5 to 56% (Ohayon, 2011; Stickley et al., 2019). Life satisfaction is defined as cognitive assessment of subjective wellbeing. Previous studies demonstrated that good sleep quality predicted higher life satisfaction (Kim and Ko, 2018; Shin and Kim, 2018). According to a nationwide cohort study in Finland, poor sleep quality was associated with a higher risk of life dissatisfaction, compared with good sleep quality [odds ratio (OR) = 2.1, 95% confidence interval (CI): 1.7–2.7] (Paunio et al., 2008). Lemola et al. using MIDUS study also reported that subjective sleep quality is related to lower subjective wellbeing among middle-aged American adults (Lemola et al., 2013). In addition, previous studies demonstrated that perceived stress and depression were related to sleep problems and life dissatisfaction (Friedman, 2016; Seo et al., 2018). A study among 307 urban African American adults suggested that anxiety and depression are independently linked to life satisfaction (Dunne et al., 2018). A prospective cohort study with 351 community-dwelling older American adults revealed that sleep disturbance acts as an independent risk factor for depression recurrence (Cho et al., 2008). A community longitudinal study of 3,636 young and middle-aged Australian adults demonstrated that self-reported sleep disturbance was significantly associated with an onset of major depressive disorder (*p* = 0.006; Batterham et al., 2012). A longitudinal study with 302 midlife women from the Study of Women's Health Across the Nation revealed that poorer sleep health is associated with higher depressive symptoms (*p* < 0.001; Bowman et al., 2021). A review conducted by Smagula et al. demonstrated that perceived stress is a major risk factor of sleep disturbance among American older adults (Smagula et al., 2016).

Overall, there is existing evidence establishing a link between sleep and life satisfaction, while depression and perceived stress are associated with a higher risk of poor sleep quality and life dissatisfaction (Glei et al., 2013; Chirinos et al., 2017). Although the associations among sleep quality, life satisfaction, depressive symptoms and perceived stress were shown in previous studies, it is still unclear how these psychosocial factors, like depressive symptoms and perceived stress, mediate the pathway through which sleep quality impacts on life satisfaction. Thus, our study used a serial mediation model to explore the serial multiple mediation effects of perceived stress and depressive symptoms on the association.

The present study examined the potential mediating effects of perceived stress and depressive symptoms on the association between sleep quality and life satisfaction. We hypothesized that poor sleep quality, perceived stress and depressive symptoms would be associated with a lower level of life satisfaction. A serial mediation model hypothesizes a causal chain linking of the mediators (perceived stress and depressive symptoms) with a specified direction flow (sleep quality → perceived stress → depressive symptoms → life satisfaction).

## Materials and Methods

### Study Data and Participants

This study used data from the Midlife in the United States (MIDUS) Study which is the first national survey of midlife development. The aims of MIDUS are to investigate the role of behavioral, psychological, and social factors in accounting for age-related variations in health and wellbeing in a national sample of Americans (National Institue on Aging., 2020). As was previously reported (Dienberg Love et al., 2010; Chen et al., 2012), participants in MIDUS 2 were at age 35–84 in 2004–2006 (MIDUS 2, *n* = 4,963), and at age 43–94 in 2013–2014 (MIDUS 3, *n* = 3,294). We used MIDUS 2, MIDUS biomarker (*n* = 1,255), and MIDUS 3 to analyze longitudinal data and examined if perceived stress and depressive symptoms mediated the association between sleep quality and life satisfaction. Study participants only were included in the present study if they were MIDUS 2 and MIDUS 3 participants and they completed the Biomarker Project (Project 4) of MIDUS 2. Participants with missing data in all relevant measurements and covariates were excluded in our study. Data for the MIDUS study was approved by the UW-Madison Education and Social/Behavioral Science Institutional Review Board.

### Measurements

#### Sleep Quality

Sleep quality was obtained from MIDUS biomarker project. A global measure of sleep quality was derived by using the Pittsburgh Sleep Quality Index (PSQI) across seven domains (Buysse et al., 1989). The PSQI is a retrospective, self-reported questionnaire containing 19-items that assess seven components of sleep and yield one global score of overall sleep quality. For this measure, participants are asked to respond to questions based on their sleep experiences over the past month. Scores are coded and summed into a global score with a possible range of 0–21. Lower global PSQI scores indicate better sleep quality. A global PSQI score >5 was defined as a poor sleep quality (Buysse et al., 1989). Several papers with MIDUS data used PSQI to report sleep quality (Owens et al., 2017; Brindle et al., 2019; Li et al., 2019).

#### Life Satisfaction

Life satisfaction as an outcome variable was obtained from MIDUS 3. Life satisfaction was measured using a five-item Self-Administered Questionnaire. Participants are asked to assess five dimensions of their lives on a scale from 0 (the worst possible) to 10 (the best possible), including life overall, work, health, relationship with spouse/partner, and relationship with children (Prenda and Lachman, 2001). The scores for the relationship with spouse/partner and the relationship with children are averaged to create one item. Then, this score is used along with the remaining three items to calculate an overall mean score. Higher scores indicate higher levels of life satisfaction and wellbeing.

#### Depressive Symptoms

Depressive symptoms were obtained from MIDUS biomarker project. Depressive symptoms were assessed using the Mood and Anxiety Symptom Questionnaire (MASQ), which is an instrument designed to measure a range of symptoms relevant to depression and anxiety, using a 5-point Likert scale (1 = not at all, 2 = little bit, 3 = moderately, 4 = quite a bit, 5 = extremely) (Buckby et al., 2007). General Distress-Depression assesses depressed/sad mood and other non-specific depressive symptoms (12 items; e.g., “felt sad,” “felt like a failure”). All 12 items on a scale range from 12 to 60. Higher scores on General Distress-Depression were reflective of higher depressive symptoms.

#### Perceived Stress

Perceived stress were obtained from MIDUS biomarker project. Subjective perceived stress was evaluated using a well-validated perceived stress scale (PSS) (Cohen et al., 1983). Ten questions are included in the PSS, which is designed to measure the extent to which participants perceive their lives as unpredictable, uncontrollable, and overloaded. Each response is coded on a five-point scale (0–4), and all 10 items range from 0 to 40 (Chaaya et al., 2010). Higher scores reflect greater perceived stress (Vigoureux et al., 2020).

#### Covariates

Several confounding variables that are linked to life satisfaction at follow-up were included as covariates in this study: age, gender, race, and life satisfaction at baseline. All of these covariates were obtained from MIDUS 2. All statistical models were multivariable-adjusted for relevant covariates.

### Statistical Analyses

The demographic variables, sleep quality, depressive symptoms, perceived stress scale and life satisfaction were described with mean, standard deviation (SD), and range, number (N), and percentage (%) as appropriate. To test the hypotheses that sleep quality, perceived stress, and depressive symptoms are associated with life satisfaction, we ran hierarchical multiple regression models with the control variables of age, gender, race and life satisfaction at baseline. All of the covariates were entered into model 1, sleep quality was added in model 2, and depressive symptoms and perceived stress entered together in model 3. Participants with missing values of sleep quality and life satisfaction were excluded in our study.

After association between sleep quality and life satisfaction was observed, a serial mediation model with four factors was applied to examine whether the association between sleep quality and life satisfaction was mediated by perceived stress and depressive symptoms. Three mediation tests were performed simultaneously. They were the triangle pathways: sleep quality → perceived stress → life satisfaction, sleep quality → depressive symptoms → life satisfaction, and the quadrangle pathway: sleep quality → perceived stress → depressive symptoms → life satisfaction. Sensitivity analysis was also conducted to detect the effects of the opposing relationship between perceived stress and depressive symptoms. The serial mediation model was analyzed using the PROCESS macro for SAS as proposed by Preacher and Hayes (Bolin, 2014; Blair, 2020).

**Figure 1:**
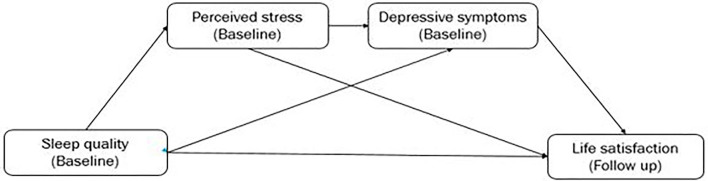
The conceptual model of serial mediation with half-longitudinal study.

Mediation analyses aim to explain how an exposure causes its putative effect on the outcome (Hayes, 2017). Ideally, all measurements should be separated in different-wave studies. However, Cole and Maxwell argue that half-longitudinal mediation may be studied in two-wave studies (Cole and Maxwell, 2003). Previous studies successfully have revealed that this statistical approach is applicable to demonstrate the role of mediators (Corlier et al., 2018; Li et al., 2018). Thus, our study applied a half-longitudinal study design to explore the effects of perceived stress (baseline) and depressive symptoms (baseline) on the association between sleep quality (baseline) and life satisfaction (follow-up). Serial multiple mediation analyses were based on 10,000 bootstrapped samples using Hayes' PROCESS. The conceptual model of a half-longitudinal study is shown in Figure 1. All of the analyses were conducted using SAS 9.4 and all statistical tests were two-sided, with *P* < 0.05 used to indicate statistical significance.

## Results

### Descriptive Statistics

The descriptive characteristics of participants are presented in Table 1. A total of 945 participants with mean age of 54.33 were included in our study. Among them, 420 (44.44%) were males and 877 (92.8%) were whites. Most participants at baseline reported a higher level of life satisfaction (mean = 7.88), which almost equals the level of life satisfaction at follow-up (mean = 7.9). Participants in our study yielded the following descriptive statistics: sleep global score (mean = 5.79); depressive symptom (mean = 18.1); and perceived stress scale (mean = 21.42).

**Table 1:** Descriptive statistics for sample (*N* =945).

**Variable**	**M (SD)**	**Range**	***N*** **(%)**
Age	54.33 (11.06)	34–83	
Gender (% male)			420 (44.44)
Race (% white)			877 (92.80)
Life satisfaction at baseline	7.80 (1.15)	3.2–10	
Sleep quality	5.79 (3.40)	0–18	
Depressive symptoms	18.10 (6.15)	12–60	
Perceived stress scale	21.42 (6.10)	10–48	
Life satisfaction	7.90 (1.28)	1–10	

In the hierarchical regression models, demographic variables (including age, gender, and race) and life satisfaction at baseline were entered into model 1. Sleep quality was added in model 2 and depressive symptoms and perceived stress were added in model 3. As shown in Table 2, after adjusting for age, gender, race, and life satisfaction at baseline, poor sleep quality was negatively associated with a higher level of life satisfaction. In addition, depressive symptoms and perceived stress were significantly associated with life satisfaction (adjusted *R*^2^ = 0.4098, *F*_change_ = 85.5, *P* < 0.01).

**Table 2:** Summary of hierarchical regression analyses for life satisfaction.

**Variable**	**Model 1**			**Model 2**			**Model 3**		
	***t***	***p***	**β**	***t***	***p***	**β**	***t***	***p***	**β**
Age	1.19	0.2333	0.0355	1.73	0.0839	0.0485	1.06	0.2877	0.0295
Gender	0.11	0.9152	0.0046	1.01	0.3147	0.0274	1.38	0.1667	0.0371
Race	−0.37	0.7084	−0.0117	−1.02	0.3101	−0.0274	−1.26	0.2068	−0.0335
Life satisfaction at baseline	21.94	<0.0001	0.6030	19.20	<0.0001	0.5632	15.81	<0.0001	0.4851
Sleep quality				−4.50	<0.0001	−0.1288	−2.35	0.0189	−0.0689
Perceived stress scale							−2.16	0.0311	−0.0810
Depressive symptoms							−3.89	0.0001	−0.1473
Adjusted *R^2^*			0.3755			0.3895			0.4098
*F* for change in *R^2^*				135.85	<0.0001		85.50	<0.0001	

### Mediation Analysis

**Figure 2:**
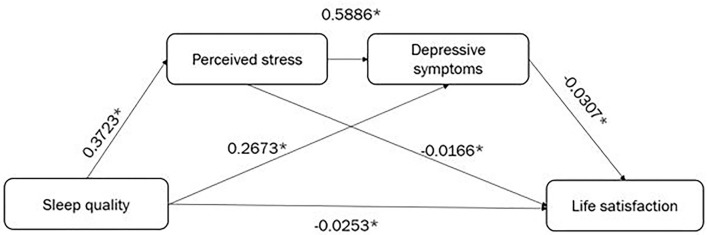
The serial mediation of perceived stress and depressive symptoms. *P < 0.05, all results are statistically significant.

Serial mediation analysis was applied to test whether the association between sleep quality and life satisfaction was mediated by perceived stress and depressive symptoms, after adjusting for all covariates. The serial models simultaneously tested three mediation pathways (see Supplementary Table 1). Figure 2 depicts the effects of the paths linking sleep quality to each mediator and life satisfaction. The positive signs of the effects indicate that higher sleep scores (poor sleep quality) are related to increased perceived stress and depressive symptoms. All indirect paths from sleep quality to life satisfaction were negative, showing the reduction in life satisfaction levels through the increase in the levels of the mediators. From the values given in Table 3 and Supplementary Table 2, perceived stress and depressive symptoms significantly mediated the relationship between sleep quality and life satisfaction, while controlling for demographic variables and life satisfaction at baseline. The direct effect of sleep quality on life satisfaction was significant (coefficient β = −0.0253, 95% CI = −0.0464–−0.0042). Meanwhile, the coefficient estimates—based on the use of 95% CI as evidence of the mediation of total indirect and indirect effects for perceived stress, depressive symptoms and the combination of perceived stress and life depressive symptoms—were calculated as follows: total indirect β = −0.0211, CI = −0.0332–−0.0118; indirect effect coefficient β (sleep quality → perceived stress → life satisfaction) = −0.0062, CI = −0.0125–−0.0008; indirect effect coefficient β (sleep quality → depressive symptoms → life satisfaction) = −0.0082, CI = −0.0154–−0.0029; indirect effect coefficient β (sleep quality → perceived stress → depressive symptoms → life satisfaction) = −0.0067, CI = −0.0123–−0.0026, respectively. The total indirect effect of sleep quality on life satisfaction accounted for in the overall model was 45.5%.

**Table 3:** Direct and indirect effects of sleep quality on life satisfaction.

**Outcome**	**β**	**BootSE**	**BootLLCI**	**BootULCI**
**Direct effect**	
Sleep quality—life satisfaction	−0.0253	0.0107	−0.0464	−0.0042
**Indirect effect**	
Sleep quality—perceived stress scale—life satisfaction	−0.0062	0.003	−0.0125	−0.0008
Sleep quality—depressive symptoms—life satisfaction	−0.0082	0.0032	−0.0154	−0.0029
Sleep quality—perceived stress scale—depressive symptoms—life satisfaction	−0.0067	0.0025	−0.0123	−0.0026

*Controlling for age, gender, race, life satisfaction at baseline*.

### Sensitivity Analysis

The sensitivity analysis of serial mediation analysis is shown in Supplementary Table 2. The opposing direction effects of perceived stress and depressive symptoms on the association between sleep quality and life satisfaction were presented in the sensitivity analysis (sleep quality → depressive symptoms → perceived stress → life satisfaction). The variable of depressive symptoms was included as the first mediator, while perceived stress was considered as the second mediator. According to the sensitivity analysis, we found that sleep quality was not associated with perceived stress (coefficient β = 0.0712, *P* = 0.138) and perceived stress, as the second mediator, did not mediate the association between sleep quality and life satisfaction.

## Discussion

To our knowledge, this is the first study using serial mediation to test the effects of psychosocial factors on the association between sleep and life satisfaction within a middle-aged population in the United States. In the present study, the mean score of sleep quality was 5.79 ± 3.4. Our study results showed that 55.13% of American adults experienced poor sleep quality, which is higher than the prevalence reported from The Sleep Foundation (SleepFoundation., 2014). According to the report from The Sleep Foundation, it is estimated that 35% of Americans have poor sleep quality, although they obtain sufficient sleep hours (SleepFoundation., 2014). The findings of our study also indicated that poor sleep quality, depressive symptoms and perceived stress were negatively associated with life satisfaction, adjusted for age, gender, race and educational level, which are consistent with previous studies. A 10-year longitudinal study from the National Survey of Midlife Development in the United States revealed that insomnia symptoms had a significant relationship with wellbeing (Karlson et al., 2013). A Nationwide Cohort study of Twins has shown that poor sleep may have direct effects on the brain, emotions, and mood, which decreases the level of life satisfaction (Paunio et al., 2008). Another population-based cross-sectional study among American adults demonstrated that good sleep quality was found to predict greater quality of wellbeing and life satisfaction (Jean-Louis et al., 2000).

Serial mediation analyses showed that perceived stress and depressive symptoms mediate the association between sleep quality and life satisfaction. The total indirect effect of sleep quality on life satisfaction through perceived stress, depressive symptoms and the combination of perceived stress and depressive symptoms accounted for the overall model was 45.5%, which indicates that mediation effects of perceived stress and depressive symptoms play important roles in the association between sleep quality and life satisfaction. Sensitivity analysis revealed that perceived stress, as the second mediator, was not associated with sleep quality directly and did not mediate the association between sleep quality and life satisfaction. Thus, poor sleep quality may lead to increased depressive symptoms, partially through increased perceived stress, which related to decreased life satisfaction. A study in China with elderly Chinese demonstrated depression plays partially mediated role on the associations of sleep duration and sleep quality with life satisfaction (Zhi et al., 2016). A cross-sectional study in Korea reported that depression was associated with sleep quality life (OR = 1.259, 95% CI 1.196–1.324, *p* < 0.001) and life satisfaction (OR = 0.881, 95% CI 0.837–0.891, *p* < 0.001) (Seo et al., 2018). A study in Nepal with mediation analysis demonstrated that depression had a significant direct effect on life satisfaction (β = −0.87, 95% CI: −1.01, −0.74) (Ghimire et al., 2018). Although few studies examine the mediating role of perceived stress on the association between sleep quality and life satisfaction, previous studies revealed the associations of sleep quality and life satisfaction with perceived stress. It is well-documented that sleep disruption can have an influence on levels of neurotransmitters and stress hormones, impairing thinking and emotional regulation (Chirinos et al., 2017; Harvardhealth., 2019; Huang and Zhu, 2020). A daily diary project from MIDUS study also revealed that daily stressor exposure predict daily wellbeing and higher daily stressor severity has been shown to be associated with lower levels of daily wellbeing (Surachman et al., 2019). Thus, perceived stress and depressive symptoms can be considered as mediators that impact the association between sleep quality and life satisfaction. At the intervention level, programs designed to improve the level of life satisfaction among adults should focus on perceived stress, depressive symptoms among middle-aged American adults.

Results and interpretations of the present study should be considered in light of several limitations. First, the study invoked a half—longitudinal study design in that the measures of sleep quality (exposure), perceived stress, and depressive symptoms (mediators) were obtained concurrently. The effects of sleep quality on perceived stress and depressive symptoms might be biased. However, several previous studies also applied mediation models, based on half-longitudinal study designs (Lyu and Agrigoroaei, 2016; Posick et al., 2019; Grossman and Gruenewald, 2020). A review on mediation models suggests that there still is progress to be made both in terms of the use of cross-sectional data, as well as the proper application of longitudinal models of mediation (O'Laughlin et al., 2018). Second, the psychosocial variables and sleep quality variables were measured by self-reported questionnaires. Therefore, there might be some self-reported bias which affects our results. Third, most participants included in our study were whites and we included all participants with complete data. The results cannot be generalized for all populations and our results may present selection bias. Forth, potential confounding factors, like marital status, may not be considered as additional covariates. Despite these limitations, this study has numerous strengths. We used longitudinal data to explore the causal relationships of sleep quality, perceived stress and depressive symptoms with life satisfaction. In addition, this is the first study that examined the serial mediation effects of perceived stress and depressive symptoms on the association between sleep quality and life satisfaction among American adults.

## Conclusion

The present study was the first to examine perceived stress and depressive symptoms as serial mediators of the relationship between sleep quality and life satisfaction. The results of the present study indicate that perceived stress followed by depressive symptoms may be important mechanisms that contribute to the positive relationship between sleep quality and life satisfaction. However, our findings do not support the contention that perceived stress and depressive symptoms operate in opposing directions in the relationship between sleep quality and life satisfaction. This causal chain should be confirmed by further longitudinal study. The findings from our study also indicated the prevention of perceived stress and depression may contribute to improving life satisfaction and wellbeing.

## Data Availability Statement

The original contributions presented in the study are included in the article/Supplementary Material, further inquiries can be directed to the corresponding author/s.

## Author Contributions

YY developed the research project, with the contribution of YC and VC. BA, KS, and DA reviewed the article. YY prepared the dataset and carried out the data analysis. All authors contributed to the article and approved the submitted version.

## Conflict of Interest

The authors declare that the research was conducted in the absence of any commercial or financial relationships that could be construed as a potential conflict of interest.

## Publisher's Note

All claims expressed in this article are solely those of the authors and do not necessarily represent those of their affiliated organizations, or those of the publisher, the editors and the reviewers. Any product that may be evaluated in this article, or claim that may be made by its manufacturer, is not guaranteed or endorsed by the publisher.
