# Supplementary material for: Serial Mediation Roles of Perceived Stress and Depressive Symptoms in the Association Between Sleep Quality and Life Satisfaction Among Middle-Aged American Adults
Source: Front Psychol. 2022 Feb 21;13:822564. doi: 10.3389/fpsyg.2022.822564 (PMC8899090; doi:10.3389/fpsyg.2022.822564)
Supplement: Supplementary file 1 [file Table_1.docx]

Supplementary Material

# Supplementary Tables

| Supplemental Table 1 Mediation analysis | | | | | | |
| --- | --- | --- | --- | --- | --- | --- |
| Variables | β | BootSE | t | P | BootLLCI | BootULCI |
| Outcome: Perceived stress scale | | | | | | |
| Sleep quality | 0.3723 | 0.0576 | 6.4639 | <.0001 | 0.2593 | 0.4854 |
| Outcome: Depressive symptoms | | | | | | |
| Sleep quality | 0.2673 | 0.0459 | 5.8239 | <.0001 | 0.1772 | 0.3574 |
| Perceived stress scale | 0.5886 | 0.0267 | 22.0238 | <.0001 | 0.5361 | 0.6410 |
| Outcome: life satisfaction | | | | | | |
| Sleep quality | -0.0253 | 0.0107 | -2.3515 | 0.0189 | -0.0464 | -0.0042 |
| Perceived stress scale | -0.0166 | 0.0077 | -2.1598 | 0.0311 | -0.0317 | -0.0015 |
| CESD: depressive symptoms | -0.0307 | 0.0079 | -3.891 | 0.0001 | -0.0462 | -0.0152 |
| Note: controlling for age, gender, race, life satisfaction at baseline | | | | | | |

| Supplemental Table 2 Sensitivity analysis | | | | | | |
| --- | --- | --- | --- | --- | --- | --- |
| Variables | β | BootSE | t | P | BootLLCI | BootULCI |
| Outcome: Depressive symptoms | | | | | | |
| Sleep quality | 0.4864 | 0.0562 | 8.6611 | 0.0000 | 0.3762 | 0.5967 |
| Outcome: Perceived stress scale | | | | | | |
| Sleep quality | 0.0712 | 0.0479 | 1.4845 | 0.1380 | -0.0229 | 0.1653 |
| Depressive symptoms | 0.6191 | 0.0281 | 22.0238 | 0.0000 | 0.5640 | 0.6743 |
| Outcome: life satisfaction | | | | | | |
| Sleep quality | -0.0253 | 0.0107 | -2.3515 | 0.0189 | -0.0464 | -0.0042 |
| Depressive symptoms | -0.0307 | 0.0079 | -3.891 | 0.0001 | -0.0462 | -0.0152 |
| Perceived stress scale | -0.0166 | 0.0077 | -2.1598 | 0.0311 | -0.0317 | -0.0015 |
| Note: controlling for age, gender, race, life satisfaction at baseline | | | | | | |
